# An unusual cause of cardiac arrest in a young infant

**DOI:** 10.1186/s12872-024-04028-1

**Published:** 2024-07-17

**Authors:** Yuhao Wu, Siyi Che, Yonggang Li

**Affiliations:** 1 Department of Cardiothoracic Surgery, Children’s Hospital of Chongqing Medical University, Chongqing, China; 2 Ministry of Education Key Laboratory of Child Development and Disorders, Chongqing Key laboratory of Pediatrics, National Clinical Research Center for Child Health and Disorders, Chongqing, China; 3 Department of Radiology, Children’s Hospital of Chongqing Medical University, Chongqing, China

**Keywords:** Anomalous coronary artery, Infant, Cardiac arrest

## Abstract

**Background:**

Anomalous aortic origin of a coronary artery from the inappropriate sinus of Valsalva (AAOCA) is a rare congenital heart lesion. It is uncommon for patients with AAOCA to present with severe symptoms at a very young age.

**Case presentation:**

We describe a very rare but critical presentation in a young infant with AAOCA that requires surgical repair and pacemaker placement. A three-month-old infant was referred because of syncope. Cardiac arrest occurred shortly after admission. The electrocardiogram indicated a complete atrioventricular block and a transvenous temporary pacemaker was implanted. A further coronary computed tomographic angiography (CTA) showed the anomalous origin of the right coronary artery from the left sinus of Valsalva. Coronary artery unroofing was performed due to an interarterial course with the intramural component, and a permanent epicardial pacemaker was implanted. The postoperative recovery was uneventful, and this patient was thriving and asymptomatic at the nine-month follow-up. However, the electrocardiogram still indicated a complete pacing rhythm.

**Conclusions:**

By timely diagnosis and treatment, this patient is successfully rescued. Although rare, AAOCA may be fatal even in infants.

**Supplementary Information:**

The online version contains supplementary material available at 10.1186/s12872-024-04028-1.

## Background

Anomalous aortic origin of a coronary artery from the inappropriate sinus of Valsalva (AAOCA) is an uncommon congenital heart lesion with a pooled prevalence ranging from 0.09 to 0.3% [1]. AAOCA may cause coronary ischemia and cardiac sudden death, especially in young athletes [2]. Patients with AAOCA may present with symptoms such as chest pain, syncope, or dyspnea. However, many cases are asymptomatic and found by routine echocardiogram coincidently. In the literature, most studies have reported surgical management of AAOCA in teenagers or adults [3–6]. It is uncommon for patients with AAOCA to present with severe symptoms at a very young age. Herein, we described a very rare but critical presentation in a young infant with AAOCA that required surgical repair and pacemaker placement simultaneously.

## Case presentation

**Fig. 1:**
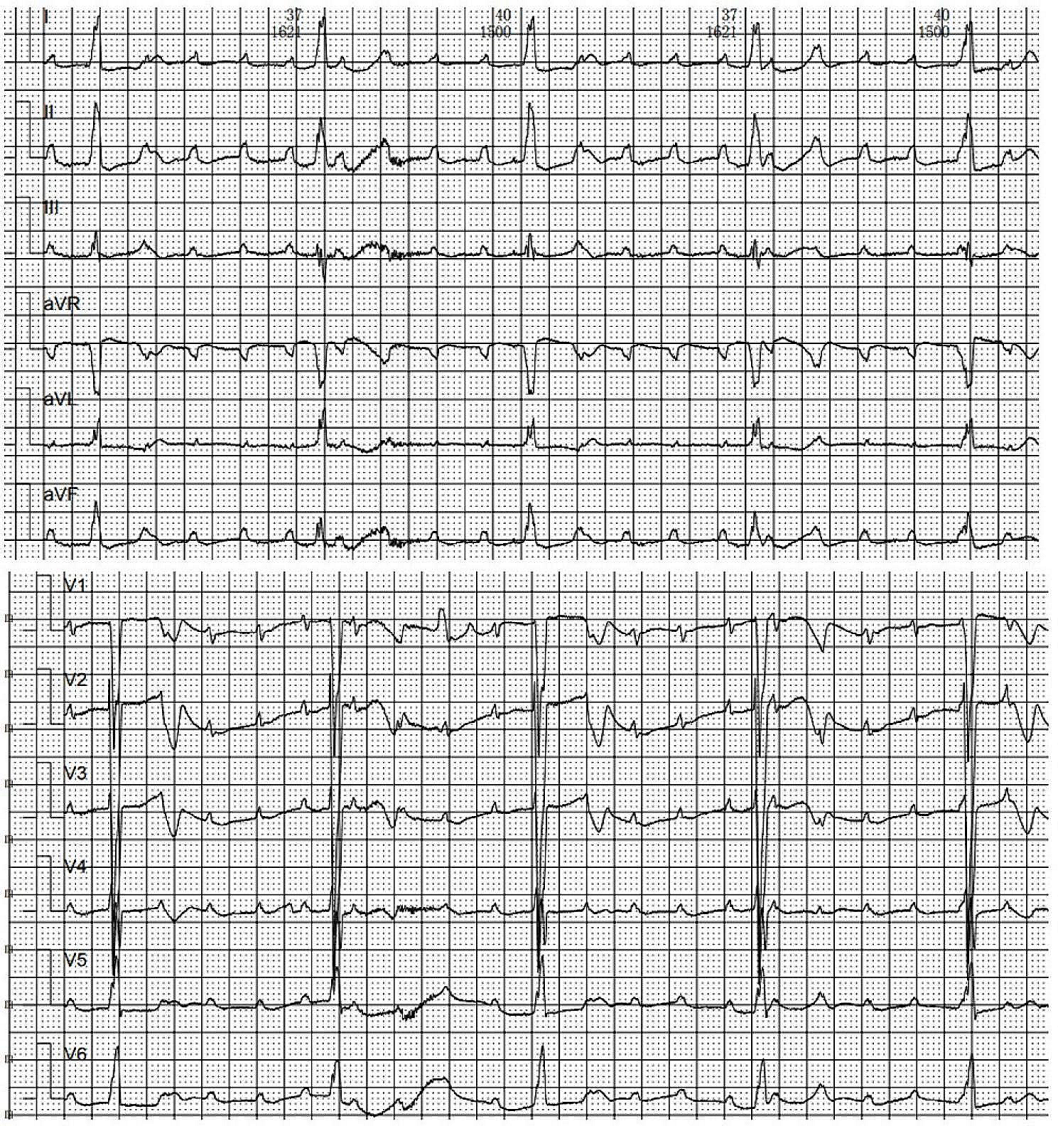
The twelve-lead electrocardiogram indicates a complete atrioventricular block with broad QRS complexes

**Fig. 2:**
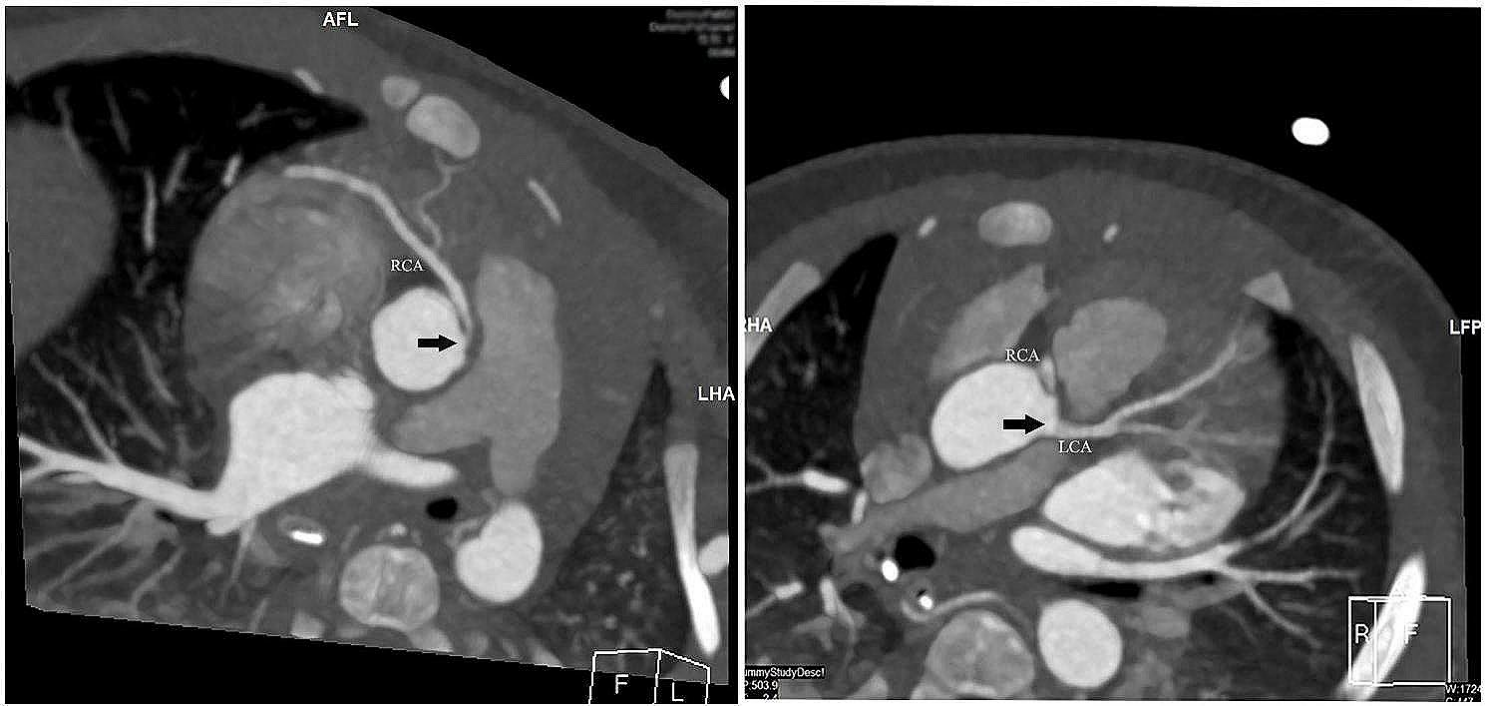
CT coronary angiography shows the anomalous origin of the RCA from the left sinus of Valsalva (Black arrow). RCA, Right coronary artery; LCA, Left coronary artery

**Fig. 3:**
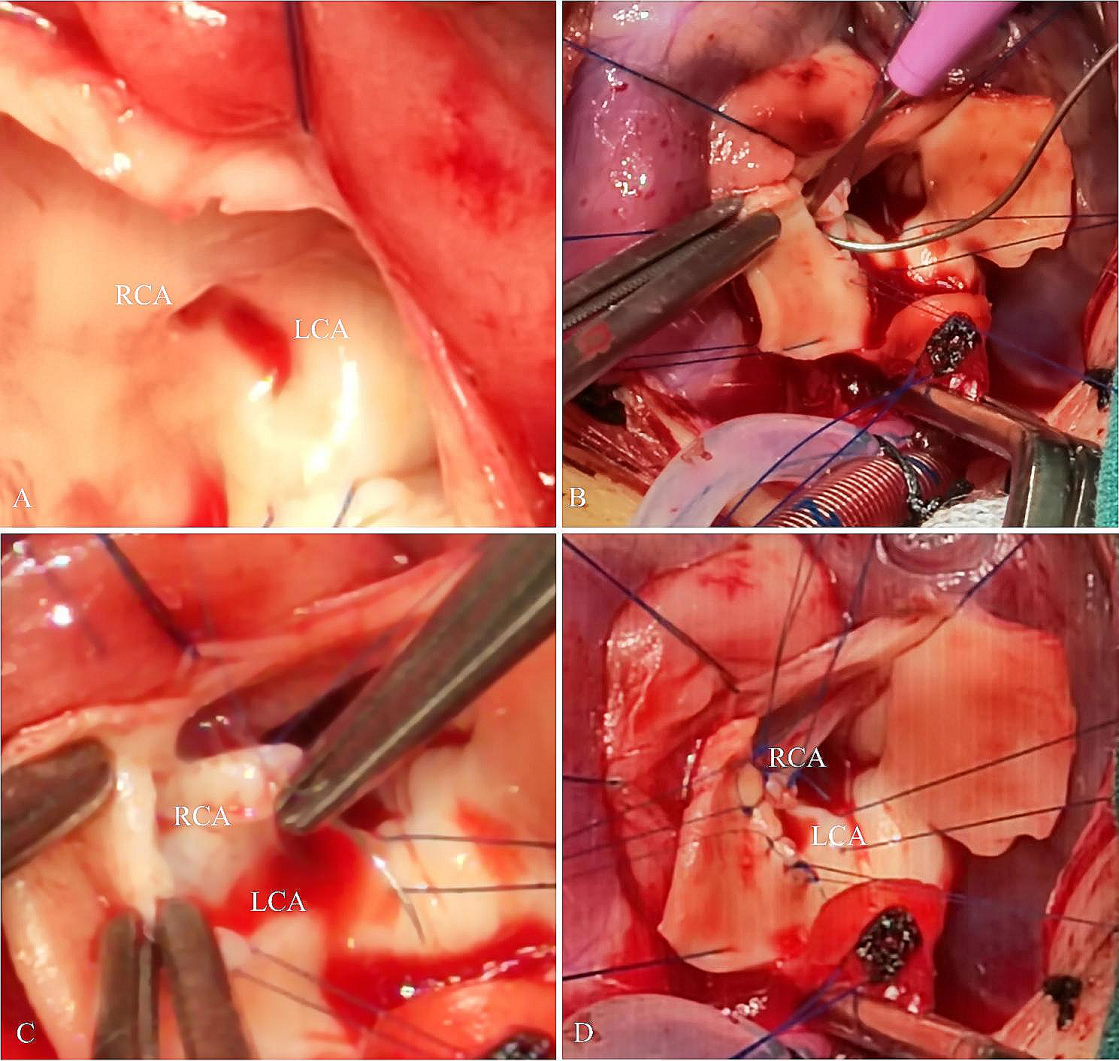
Surgical images during the operation. **A**. RCA arises from the left sinus of Valsalva with an intramural segment; **B**, **C**, and **D**. Procedures of coronary artery unroofing. RCA, Right coronary artery; LCA, Left coronary artery

A three-month-old infant was referred to our institution because of syncope one day prior. Her syncope occurred suddenly and lasted for two minutes with cyanosis and seizures. She had no diseases or operations in the past. Her heart rhythm was normal at birth with a heart rate of 160 beats/minute. Her mother had no illness during the perinatal period. Physical examinations revealed that the heart rate was 40 beats/minute, blood pressure was 64/32 mmHg, respiratory rate was 49 times/minute, and oxygen saturation was 98%. Auscultation revealed a 3/6 systolic murmur at the parasternal spaces. Abdominal palpation revealed hepatomegaly. Unfortunately, cardiac arrest occurred shortly after admission. After cardiopulmonary resuscitation and defibrillation, she was transferred to the intensive care unit (ICU). During the hospitalization in the ICU, her electrocardiogram indicated a complete atrioventricular block (AVB) with broad QRS complexes. The ventricular rate was 39 beats/minute (Fig. 1). Blood tests showed an elevation of cardiac enzymes and troponin. The echocardiogram revealed a significantly enlarged left ventricle, a mildly enlarged left atrium, and mild-to-moderate regurgitation of the mitral and tricuspid valves. Despite careful screening, transthoracic echocardiogram showed a negative result for coronary arteries. Emergency temporary pacemaker implantation was performed by the cardiologists. To clarify other potential structural abnormalities, a further coronary computed tomographic angiography (CTA) was performed. Coronary CTA showed the anomalous origin of the right coronary artery from the left sinus of Valsalva (Fig. 2). The diagnosis of AAOCA was confirmed. Therefore, surgical repair was considered. During the operation, we found that the right coronary artery (RCA) arose from the left sinus of Valsalva with an intramural segment (Fig. 3A). Additionally, the proximal course of the RCA was interarterial between the pulmonary artery and the aorta. The top of inter-coronary commissure was below the intramural segment. We performed coronary artery unroofing for this patient (Fig. 3B, C, and D). Since the length of the interarterial course was limited, we did not perform pulmonary artery translocation. A permanent epicardial pacemaker was implanted simultaneously. The postoperative recovery was uneventful, and she was discharged ten days following surgery. This patient was thriving and asymptomatic at the nine-month follow-up. The echocardiogram showed no signs of narrowing of coronary arteries. The left ventricular ejection fraction was 62%. However, the electrocardiogram still indicated a complete pacing rhythm.

## Discussion and conclusions

Although the vast majority of cases with AAOCA are adolescents without symptoms, a previous study has shown an elevated risk of sudden death with interarterial anomalous left coronary artery (ALCA) and right coronary artery (ARCA) [1]. In the literature, sudden cardiac death has been described in 30% of patients with ARCA and 70% of patients with ALCA [7].

Several imaging modalities have been adopted for the diagnosis of AAOCA. Transthoracic echocardiogram (TTE) is a non-invasive and rapid technique used to evaluate the location of the coronary ostia and coronary course. However, TTE is very limited in diagnosing AAOCA correctly [8]. Thankavel et al. [9] reported a novel echocardiographic screening method which only improved the detection rate of anomalous origin of a coronary artery from 0.02 to 0.22%. In our case, TTE did not detect the anomalous coronary artery, although careful screening of the coronary arteries was performed. The American College of Cardiology/American Heart Association guidelines recommend coronary CTA and magnetic resonance as the Class I-indicated tests for the diagnosis of AAOCA [10]. Invasive coronary angiography is also limited in depicting coronary vessels, and nearly half of patients refer to coronary CTA after a prior coronary angiography in a previous cohort [11].

Recommendations for AAOCA management remain debated regarding the indications for surgical repair of ALCA and ARCA with interarterial course. Surgery is suggested for interarterial ALCA and ARCA with the presence of ischemia. On the contrary, conservative approaches may be considered in interarterial ARCA without ischemia or narrowing [1]. Current evidence has shown that surgery is safe and effective in the treatment of AAOCA with low morbidities and mortalities. Surgical methods mainly include coronary artery unroofing, reimplantation, coronary bypass, or neo-ostia creation.

Congenital AVB is rare in children. The autoimmune process is an important cause of AVB in neonates and children [12]. Maternal anti-Ro/SSA may enter the fetal circulation and impair the conduction system [13]. Based on the preoperative electrocardiogram, ST elevation or myocardial infarction was not observed. Therefore, congenital AVB with incidental anomalous RCA might be suspected. However, this patient had no illness since birth, and her mother was also negative for anti-Ro/SSA. As a result, congenital AVB was less likely in this patient.

In conclusion, this case demonstrates the first and youngest infant with AAOCA that requires surgical repair and pacemaker placement in the literature. By timely diagnosis and treatment, this patient is successfully rescued. Although rare, AAOCA may be fatal even in young infants.

### Electronic supplementary material

Below is the link to the electronic supplementary material.

Supplementary Material 1

## Data Availability

The data underlying this article will be shared on reasonable request to the corresponding author.

## Abbreviations

ALCA: Anomalous left coronary artery
ARCA: Anomalous right coronary artery
CTA: Coronary computed tomographic angiography
AVB: Atrioventricular block
TTE: Transthoracic echocardiogram
ICU: Intensive care unit
AAOCA: Anomalous aortic origin of a coronary artery from the inappropriate sinus of Valsalva
RCA: Right coronary artery
LCA: Left coronary artery
